# Tirzepatide in Metabolically Dysfunctional‐Associated Steatohepatitis (MASH): A Bibliometric and Evidence‐Based Review

**DOI:** 10.1155/jdr/1853763

**Published:** 2026-04-10

**Authors:** Ileana Pantea, Angela Repanovici

**Affiliations:** ^1^ Transilvania University of Brasov, Brasov, Romania, unitbv.ro

**Keywords:** fibrosis, GIP/GLP-1 agonists, MASH, MASLD, tirzepatide, type 2 diabetes

## Abstract

**Purpose:**

Metabolically‐dysfunction‐associated steatohepatitis (MASH) is the progressive form of metabolic dysfunction‐associated steatotic liver disease (MASLD) and is strongly linked to obesity and type 2 diabetes mellitus (T2D). Tirzepatide, a dual glucose‐dependent insulinotropic polypeptide (GIP)/glucagon‐like peptide‐1 (GLP‐1) receptor agonist, has emerged as a promising therapeutic option due to its profound metabolic effects and potential hepatic benefits. This study integrates bibliometric mapping with current clinical evidence to evaluate tirzepatide’s role in MASLD/MASH.

**Methods:**

A bibliometric search was conducted in the Web of Science Core Collection (2018–2025) using predefined keywords related to tirzepatide and metabolic liver disease. Twenty‐five full‐text publications—including randomized controlled trials, post hoc analyses, meta‐analyses, mechanistic reviews, and international guidelines—were systematically reviewed. Co‐occurrence networks were generated using VOSviewer.

**Results:**

Scientific output on tirzepatide and MASLD/MASH has increased rapidly since 2020, with thematic clusters centered on metabolic regulation, hepatic inflammation, and fibrosis. Clinical evidence shows that tirzepatide induces substantial weight loss, improves glycaemic control, and reduces hepatic biomarkers such as ALT, AST, K 18, and Pro C3. The SYNERGY‐nonalcoholic steatohepatitis (NASH) trial demonstrated high rates of MASH resolution without fibrosis worsening and meaningful fibrosis regression at 52 weeks. Network meta‐analyses position tirzepatide among the most effective therapies currently available. Recent EASL–EASD–EASO guidelines recommend tirzepatide for MASLD patients with obesity, T2D, or cardiometabolic risk.

**Conclusions:**

Tirzepatide combines potent metabolic effects with significant hepatic improvements, positioning it as a promising therapy for MASLD/MASH. Although long‐term and phase III data are still needed, current evidence supports tirzepatide as a key component of modern metabolic liver disease management.

## 1. Introduction

Metabolic dysfunction‐associated steatohepatitis (MASH), previously known as nonalcoholic steatohepatitis (NASH), represents the progressive and clinically significant form of metabolic dysfunction‐associated steatotic liver disease (MASLD). It is characterized by hepatic steatosis, inflammation, hepatocellular injury, and varying degrees of fibrosis, ultimately predisposing patients to cirrhosis, hepatocellular carcinoma, and liver‐related mortality [1, 2]. The global rise in obesity, insulin resistance, and type 2 diabetes mellitus (T2D) has led to a parallel increase in MASLD/MASH prevalence, making it one of the most common chronic liver diseases worldwide and a major public health concern [2–5].

MASLD also exhibits a close and bidirectional relationship with cardiovascular disease (CVD), driven by shared pathophysiological mechanisms such as systemic inflammation, endothelial dysfunction, insulin resistance, dyslipidaemia, and prothrombotic states. These pathways contribute simultaneously to hepatocellular injury, visceral adiposity, fibrosis progression, and vascular damage. Cardiovascular complications—including myocardial infarction, stroke, arrhythmias, and heart failure—often precede clinically apparent liver‐related events, underscoring the systemic nature of MASLD. Recent evidence highlights particularly strong associations with heart failure with preserved ejection fraction (HFpEF) and atherosclerotic CVD. A bidirectional interaction is also recognized: heart failure may induce congestive hepatopathy, while advanced hepatic fibrosis can contribute to cirrhotic cardiomyopathy. These insights reinforce the need for therapeutic strategies that address both hepatic and cardiometabolic dysfunction. Recent comprehensive reviews further support this interconnected pathophysiology [6–8].

Despite extensive research, effective pharmacological treatments for MASH remain limited. Lifestyle modification remains the cornerstone of management, yet long‐term adherence and sustained histological improvement are difficult to achieve [1]. Several investigational agents have shown partial benefits, but many have failed to demonstrate consistent improvements in both steatohepatitis and fibrosis [9–11]. This therapeutic gap has intensified interest in metabolic agents capable of addressing the underlying cardiometabolic dysfunction that drives disease progression.

Tirzepatide, a dual agonist of the glucose‐dependent insulinotropic polypeptide (GIP) and glucagon‐like peptide‐1 (GLP‐1) receptors, has emerged as a promising candidate due to its profound effects on weight reduction, glycaemic control, insulin sensitivity, and lipid metabolism [12–15]. Early clinical studies demonstrated improvements in hepatic biomarkers such as ALT, AST, K 18, and PRO‐C3 among patients with T2D [16]. More recently, the SYNERGY NASH trial provided histological evidence of MASH resolution and fibrosis improvement in patients treated with tirzepatide [17–19]. These findings position tirzepatide at the intersection of metabolic and hepatologic therapy, with the potential to modify disease progression.

In addition to synthesizing clinical and mechanistic evidence, a bibliometric approach was selected to capture the rapid evolution of scientific output following the 2024 nomenclature transition from NAFLD/NASH to MASLD/MASH. Formerly termed nonalcoholic fatty liver disease (NAFLD), this condition has been reclassified as MASLD to highlight its underlying metabolic basis rather than defining it by the absence of alcohol consumption. This shift underscores the significant role of insulin resistance, obesity, and cardiometabolic dysfunction in disease pathogenesis. In the present review, historical terms (NAFLD and NASH) are preserved when referring to earlier studies, while recognizing that these entities conceptually align with current MASLD and MASH. Epidemiological evidence based on studies using the former NAFLD/NASH criteria—consistent with MASLD/MASH—indicates that more than 70% of individuals with T2D have hepatic steatosis, and up to 30% progress to steatohepatitis with ballooning, lobular inflammation, and varying degrees of fibrosis. Incorporating a bibliometric analysis, therefore, allows us to map how research themes have shifted in response to this paradigm change and to contextualize tirzepatide within the evolving landscape of metabolic liver disease.

The purpose of this study is to integrate bibliometric mapping with current clinical and preclinical evidence to evaluate the therapeutic potential of tirzepatide in MASLD/MASH.

The specific objectives are to:

1. characterize research trends and thematic clusters related to tirzepatide and MASH using bibliometric analysis;
2. summarize clinical evidence regarding tirzepatide’s metabolic and hepatic effects;
3. evaluate histological outcomes from recent clinical trials, including fibrosis and steatohepatitis resolution;
4. compare tirzepatide with other emerging therapies for MASH;
5. assess its positioning in current international guidelines and implications for clinical practice [20, 21].

Significance is as follows:

By combining bibliometric insights with evidence synthesis, this study provides a comprehensive evaluation of tirzepatide as a potential disease‐modifying therapy for MASH. Understanding its role is essential for clinicians managing patients with obesity, T2D, and metabolic liver disease and for guiding future research and therapeutic development.

## 2. Materials and Methods

### 2.1. Bibliometric Search

**Figure 1:**
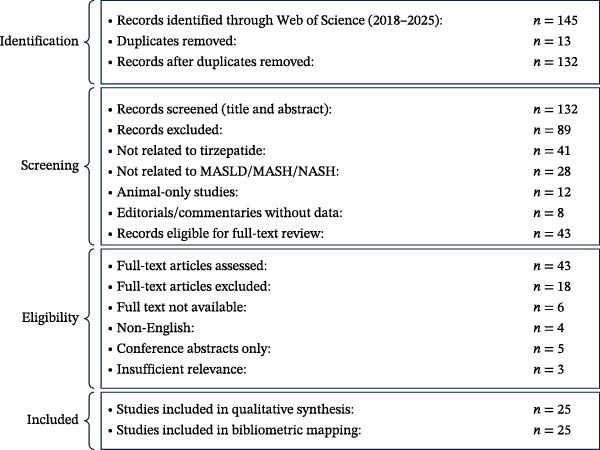
PRISMA flow diagram for bibliometric study selection.

The Web of Science Core Collection was searched for publications from 2018 to 2025 using predefined keywords related to tirzepatide, MASLD, MASH, NASH, fibrosis, GLP‐1, and GIP. After removing duplicates, 132 records were screened by title and abstract. A total of 43 full‐text articles were assessed for eligibility, and 25 studies met the inclusion criteria and were incorporated into the bibliometric mapping and qualitative synthesis (Figure 1).

The discrepancy between the 145 records initially identified and the 132 records shown in the PRISMA diagram is due to the automatic removal of 13 duplicate entries by the Web of Science interface during the export process. Thus, 145 records were retrieved, 13 duplicates were excluded, and 132 unique records proceeded to screening.

Web of Science was selected as the primary database because it provides standardized indexing, high‐quality citation metadata, and full compatibility with VOS viewer, which requires structured bibliometric fields for accurate co‐occurrence mapping. Its comprehensive coverage of high‐impact journals in hepatology, endocrinology, and metabolic research makes it particularly suitable for mapping emerging therapeutic landscapes.

We acknowledge that restricting the search to a single database may introduce selection bias, as Scopus and PubMed index additional journals and conference proceedings. This potential database bias is addressed in the Limitations section, and future bibliometric analyses may benefit from multidatabase integration to enhance coverage and reduce selection bias.

A bibliometric search was conducted in the Web of Science Core Collection (2018–2025) using the keywords “tirzepatide,” “NASH,” “MASH,” “MASLD,” “fibrosis,” “GLP‐1,” “GIP,” and “steatohepatitis.” The search strategy was informed by prior bibliometric and therapeutic landscape analyses in MASLD/MASH [6, 9, 18, 22].

The complete search strategy used in Web of Science was as follows:

^∗^TS = (“tirzepatide” OR “GIP/GLP‐1” OR “dual incretin”) AND (“MASLD” OR “MASH” OR “NASH” OR “NAFLD” OR “steatohepatitis” OR “fibrosis”)  ^∗∗^.

Boolean operators were applied across title, abstract, author keywords, and Keywords Plus fields. No filters were applied for study design or article type at the initial search stage.

### 2.2. Inclusion Criteria

Eligible publications are included as follows:

• randomized controlled trials evaluating tirzepatide in metabolic or hepatic disease [16, 17];
• post hoc analyses of clinical trials [16];
• systematic reviews and meta‐analyses [21];
• mechanistic and clinical reviews on incretin therapies and MASLD/MASH [3–7, 9, 10, 12, 13, 15–19, 22–27];
• international clinical practice guidelines [20, 28].

Conference abstracts, editorials without data, and non‐English publications were excluded.

Non‐English publications were excluded to ensure consistency in terminology and because VOSviewer relies on English‐language metadata for accurate co‐occurrence mapping. Including non‐English records would have introduced heterogeneity in keyword extraction and reduced comparability across studies. We acknowledge that this restriction may introduce language bias, and this limitation is addressed in the discussion.

### 2.3. Analytical Tools

Co‐occurrence networks were generated using VOSviewer, a validated tool for mapping scientific terminology in metabolic and hepatic research [9, 24].

Study selection followed a two‐step process. First, two independent reviewers screened all titles and abstracts to identify potentially eligible studies. The same reviewers then assessed full texts of all selected records independently. Any disagreements were resolved through discussion and consensus. Two reviewers using a standardized template to ensure accuracy and reproducibility also performed data extraction independently.

A quantitative synthesis or meta‐analysis was not performed because the included studies were highly heterogeneous in design, population characteristics, endpoints, and outcome reporting. The dataset comprised randomized trials, post hoc analyses, mechanistic reviews, and guideline documents, which precluded statistical pooling. Given this heterogeneity, a narrative evidence synthesis was deemed the most appropriate approach to accurately summarize current knowledge.

## 3. Results

### 3.1. Bibliometric Findings

**Figure 2:**
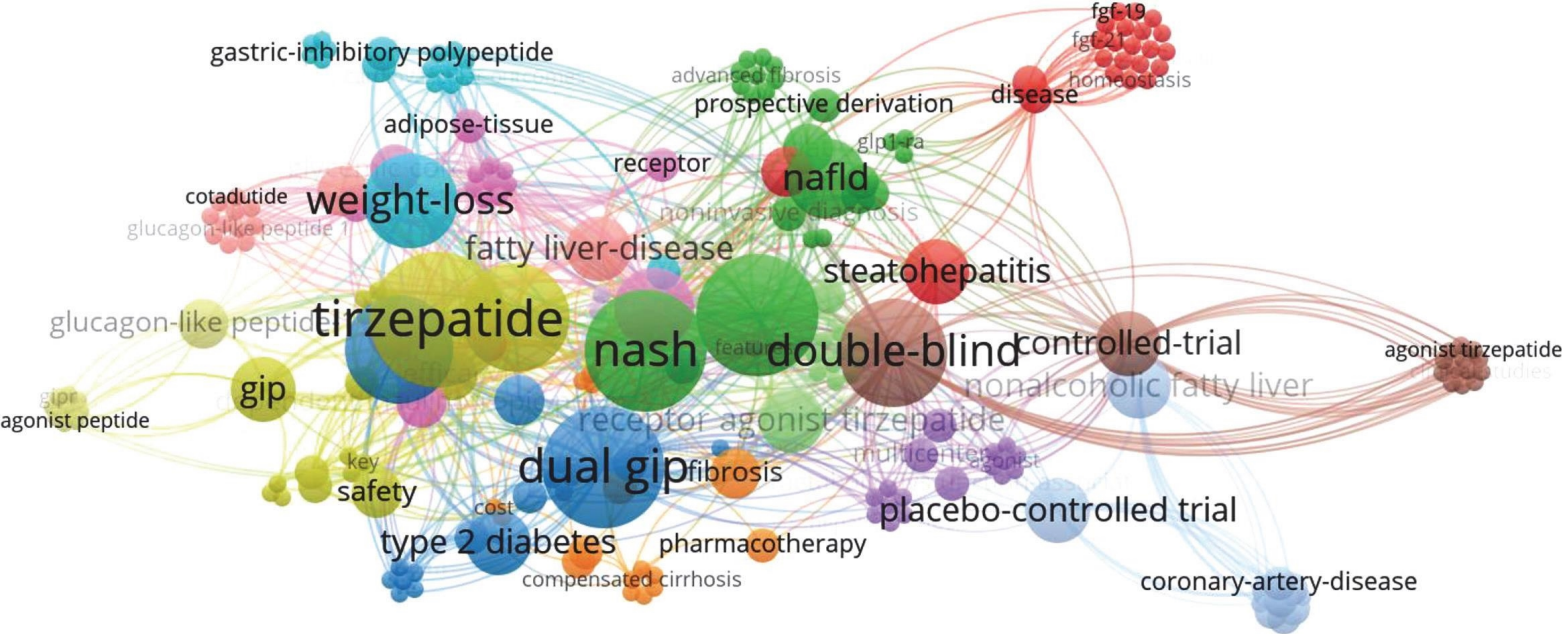
Network visualization of keyword co‐occurrence in tirzepatide–MASLD/MASH research (2018–2025).

The bibliometric analysis (Figure 2) identified a rapid increase in publications related to tirzepatide and MASLD/MASH beginning in 2020, coinciding with early biomarker studies [14] and the expansion of incretin‐based therapies [12, 15, 20].

Three major thematic clusters emerged as follows:

1. Metabolic cluster: weight loss, T2D, GLP‐1 receptor agonists, and insulin resistance—reflecting tirzepatide’s origins as a metabolic therapy [12–15].
2. Hepatic cluster: fibrosis, steatohepatitis, adipose tissue, ALT, and AST—consistent with emerging evidence of hepatic benefits [7, 8, 16, 17, 27, 29].
3. Clinical trial cluster: controlled trial, placebo‐controlled, and phase 2—driven by the SYNERGY NASH trial [17].

Tirzepatide appeared as a central node linking metabolic and hepatic domains, confirming its dual therapeutic relevance.

A more granular examination of the VOSviewer map reveals that the density of connections between tirzepatide and fibrosis‐related terms (e.g., “fibrosis,” “Pro‐C3,” “stellate cells”) has increased markedly since 2022. This pattern illustrates a rapid shift in the scientific focus from glycemic control and weight loss toward structural liver repair and antifibrotic mechanisms. The hepatic cluster shows high‐intensity linkages, indicating that tirzepatide is now frequently studied in the context of steatohepatitis activity, fibrogenesis, and histological endpoints. In contrast, the metabolic cluster remains broader but less densely interconnected, reflecting tirzepatide’s established role in metabolic regulation.

To contextualize the intellectual landscape of this emerging field, we additionally identified the most influential contributors. Table S1 (Supporting Information) summarizes the top 10 most cited authors and the top contributing journals in tirzepatide–MASLD/MASH research, providing insight into the leading voices shaping current scientific discourse.

In Figure 2, node size reflects keyword frequency, while color indicates cluster membership. Thicker connecting lines represent stronger co‐occurrence relationships. Abbreviations are defined in the figure for clarity. The figure represents a co‐occurrence map of scientific terms in the medical and pharmaceutical fields, with a focus on tirzepatide, NASH (nonalcoholic steatohepatitis), and associated metabolic diseases. Its interpretation is as follows:

Nodes and connections: each node (circle) corresponds to a medical or pharmaceutical term (e.g., tirzepatide, NASH, and T2D). The size of the node reflects its frequency in the scientific literature—the larger the node, the more frequently the term appears. The connecting lines indicate semantic relationships or co‐occurrences between terms within published studies. For example, tirzepatide is linked to weight loss, T2D, and NASH, illustrating their frequent joint appearance in research articles.

Central and relevant terms: tirzepatide appears as a central node, connected to GLP‐1, dual GIP, weight loss, and T2D, highlighting its role as an innovative therapeutic agent. NASH and NAFLD, metabolic liver diseases associated with obesity and diabetes, cluster with terms such as fibrosis, steatohepatitis, and adipose tissue. The presence of “controlled trial” and “placebo‐controlled trial” reflects the contribution of rigorous clinical studies. Terms such as “GLP‐1 receptor agonist” and “gastric inhibitory polypeptide” emphasize the pharmacological mechanisms relevant to tirzepatide.

Implications for research: The map indicates that tirzepatide is extensively studied in the context of T2D, weight reduction, and NASH therapy. The prominence of terms such as fibrosis and adipose tissue underscores the growing scientific interest in the metabolic and hepatic effects of this treatment. Such co‐occurrence patterns provide a valuable foundation for designing search strategies in Web of Science and for identifying emerging research themes.

The strong connectivity of terms like fibrosis and adipose tissue highlights the increasing focus on understanding tirzepatide’s impact beyond glycaemic control and weight loss. Their frequent association with NASH and NAFLD suggests that current research explores its potential effects on hepatic inflammation, fat accumulation, and fibrosis regression. This interconnectedness within the bibliometric network supports an integrative perspective in which tirzepatide is examined as a multifunctional therapeutic agent for complex metabolic liver diseases.

### 3.2. Clinical Evidence

#### 3.2.1. Metabolic Outcomes

Across multiple clinical trials, tirzepatide is demonstrated as follows:

• substantial weight reduction, often ≥20% in obesity studies [14, 30];
• significant HbA1c reduction in T2D [13, 22, 26];
• improvements in lipid profile and insulin sensitivity [12, 15, 20].

These metabolic effects are known mediators of MASLD/MASH improvement [9–11, 30, 31].

#### 3.2.2. Hepatic Biomarkers

In a post hoc analysis of T2D patients, tirzepatide is significantly reduced as follows:

• ALT and AST;
• K 18 and Pro C3;
• hepatic fat content and increased adiponectin [16].

These biomarkers correlate with steatohepatitis activity and fibrogenesis [4, 11].

#### 3.2.3. Histological Outcomes

The SYNERGY NASH phase 2 trial is demonstrated as follows:

• 60%–73% MASH resolution without fibrosis worsening;
• significant fibrosis regression;
• consistent effects across subgroups (sex, age, BMI, and T2D status) [17].

This represents one of the strongest histological responses reported for an incretin‐based therapy.

#### 3.2.4. Comparative Evidence

A 2025 network meta‐analysis comparing 29 pharmacological therapies for MASH found tirzepatide among the most effective agents for the following:

• MASH resolution and
• fibrosis regression [21].

Other reviews similarly position tirzepatide alongside FGF21 and FXR agonists as leading candidates in the therapeutic landscape [3, 9–11].

Beyond comparative efficacy, the mechanisms of action of tirzepatide, FGF21 analogs, and FXR agonists differ substantially and may guide therapeutic selection in clinical practice. Tirzepatide exerts its effects primarily through profound weight loss, appetite suppression, and improvements in insulin sensitivity, which collectively reduce hepatic steatosis and inflammation. In contrast, FGF21 analogs increase thermogenesis and energy expenditure, enhance lipid oxidation, reduce triglycerides and improve insulin sensitivity even in the absence of major weight loss. FXR agonists act directly on bile acid signaling pathways, reducing hepatic steatosis, improving lipid metabolism, and attenuating inflammation, although their impact on body weight is modest.

These mechanistic distinctions highlight that tirzepatide may be preferred in patients with obesity and T2D, FGF21 analogs in those with severe dyslipidaemia or metabolic syndrome, and FXR agonists in patients with MASH and prominent lipid dysregulation. A comparative summary of these therapeutic classes is provided in Tables 1 and 2.

**Table 1:** Comparative mechanisms and clinical profiles of tirzepatide, FGF21 analogs, and FXR agonists.

Feature	Tirzepatide	FGF21 analogs	FXR agonists
Weight loss	High	Moderate	Minimal
Appetite suppression	✓	✗	✗
Energy expenditure	✗	✓	✗
Glucose control	Strong	Moderate	Moderate
Lipids and liver fat	Good (via weight loss)	Strong	Strong
Primary mechanism	Appetite suppression, insulin sensitivity	Thermogenesis, lipid oxidation, metabolic regulation	Bile acid signaling, lipid metabolism
Best use case	Obesity + T2D + MASLD	Metabolic syndrome + dyslipidaemia + MASLD	MASH with lipid dysregulation

**Table 2:** Comparative summary of major tirzepatide trials: Sample size, outcomes, and safety.

Trial	Population	Sample size (tirzepatide arm)	Primary outcomes	Key efficacy findings	Key safety findings
SYNERGY‐NASH (Phase II)	Biopsy‐proven MASH, F2–F3	~190	NASH resolution, fibrosis improvement	NASH resolution ~40%–60%; ≥1‐stage fibrosis improvement ~45%–55%	GI AEs most common; nausea/diarrhea; discontinuation ~6%–10% depending on dose
SURMOUNT‐1	Obesity without diabetes	~1900	Weight loss	Mean weight loss 15%–20%	GI AEs; discontinuation 4%–7% vs 2%–3% placebo
SURPASS‐1/2	Type 2 diabetes	~1500	HbA1c reduction, weight loss	HbA1c ↓ ~2%–2.5%; weight ↓ 5%–10%	GI AEs; low discontinuation at lower doses
SURPASS‐3/5	T2D with higher metabolic risk	~1500	Glycemic control, liver enzymes	Similar glycemic and weight effects; ALT/AST improvements	GI AEs; rare serious events; discontinuation modest

### 3.3. Guideline Integration

The 2024 EASL–EASD–EASO guidelines recommend tirzepatide for MASLD patients with the following:

• T2D;
• obesity;
• cardiometabolic risk factors [27, 28, 32].

This reflects a paradigm shift toward metabolic therapies as first‐line interventions in MASLD.

## 4. Discussion

This integrated bibliometric and evidence‐based analysis highlights tirzepatide as one of the most promising therapeutic agents for MASLD/MASH. Its dual incretin mechanism—simultaneous activation of GIP and GLP‐1 receptors—produces synergistic metabolic effects that extend beyond glycemic control. These effects include substantial weight reduction, improved insulin sensitivity and favorable changes in lipid metabolism, all of which are central drivers of MASLD pathogenesis.

Across Phase II and III programs, tirzepatide has demonstrated a generally favorable safety and tolerability profile, consistent with other incretin‐based therapies. Gastrointestinal adverse events—primarily nausea, diarrhea, vomiting, and decreased appetite—represent the most frequently reported side effects. These events typically occur during the dose‐escalation phase, are dose‐dependent, and tend to diminish over time as patients adapt to treatment.

Discontinuation rates due to adverse events vary by dose: ~4%–7% in weight‐management trials and up to ~10% at the highest 15 mg dose, compared with ~2%–3% in placebo groups. Most discontinuations are attributable to gastrointestinal symptoms. Serious adverse events remain uncommon, and no new safety signals have emerged in metabolic or liver‐focused studies to date. Overall, tirzepatide’s safety profile is considered acceptable when used with appropriate monitoring and gradual dose titration.

The bibliometric mapping confirms tirzepatide’s emerging role at the intersection of metabolic and hepatic research. The clustering of terms such as fibrosis, steatohepatitis, adipose tissue, and insulin resistance reflects a growing scientific interest in the drug’s potential to modify both metabolic and hepatic pathways. The central positioning of tirzepatide within the co‐occurrence network underscores its relevance across multiple research domains.

Clinical evidence reinforces these findings. Biomarker improvements in ALT, AST, K 18, and Pro‐C3 suggest meaningful reductions in hepatocellular injury and fibrogenesis. The SYNERGY‐NASH trial provides the strongest histological evidence to date, demonstrating high rates of MASH resolution and fibrosis regression—outcomes that exceed those reported for earlier incretin therapies. Comparative analyses further position tirzepatide among the most effective agents currently under investigation for MASH.

To facilitate comparison across key tirzepatide trials, we summarized sample size, main liver and metabolic outcomes, and adverse events in a unified table (Table 3). This overview highlights both the magnitude of benefit and the safety profile across different clinical settings.

**Table 3:** Summary of key tirzepatide clinical trials: sample size, efficacy, and safety.

Trial	Population	Sample size (tirzepatide arm)	Primary outcomes	Key efficacy findings	Key safety findings
SYNERGY‐NASH (Phase II)	Biopsy‐proven MASH, F2–F3	~190	NASH resolution, fibrosis improvement	NASH resolution ~40%–60%; ≥1‐stage fibrosis improvement ~45%–55%	GI AEs most common; nausea/diarrhea; discontinuation ~6%–10% depending on dose
SURMOUNT‐1	Obesity without diabetes	~1900	Weight loss	Mean weight loss 15%–20%	GI AEs; discontinuation 4%–7% vs 2%–3% placebo
SURPASS‐1/2	Type 2 diabetes	~1500	HbA1c reduction, weight loss	HbA1c ↓ ~2%–2.5%; weight ↓ 5%–10%	GI AEs; low discontinuation at lower doses
SURPASS‐3/5	T2D with higher metabolic risk	~1500	Glycemic control, liver enzymes	Similar glycemic and weight effects; ALT/AST improvements	GI AEs; rare serious events; discontinuation modest

The integration of tirzepatide into the 2024 EASL–EASD–EASO guidelines marks a paradigm shift toward metabolic therapies as first‐line interventions in MASLD. This reflects a broader recognition that metabolic dysfunction is a primary driver of disease progression and that therapies targeting weight, insulin resistance, and adipose‐tissue inflammation may offer the most comprehensive benefits.

This integrated analysis highlights tirzepatide as a promising therapeutic option for MASLD/MASH. Its dual incretin mechanism provides synergistic metabolic effects that translate into hepatic benefits, consistent with mechanistic models of MASLD pathogenesis [4, 5, 9, 11, 12, 18].

Weight loss, improved insulin sensitivity, and reduction of hepatic steatosis appear to mediate much of the histological improvement observed in clinical trials [14, 30]. Biomarker reductions in ALT, AST, K 18, and Pro C3 further support tirzepatide’s impact on hepatocellular injury and fibrogenesis [16].

The SYNERGY NASH trial provides the strongest evidence to date, demonstrating high rates of MASH resolution and fibrosis improvement [17]. These results exceed those reported for earlier incretin therapies such as semaglutide, aligning with reviews suggesting that dual GIP/GLP‐1 agonism may offer superior metabolic and hepatic benefits [15, 20, 27].

While the SYNERGY‐NASH findings are highly encouraging, they remain Phase II data and therefore exploratory. The trial was not powered to assess long‐term clinical outcomes such as progression to cirrhosis, hepatic decompensation, or mortality. Consequently, although tirzepatide demonstrated meaningful histologic improvements—including NASH resolution and potential fibrosis regression—these results should be interpreted as proof of concept rather than definitive evidence of disease modification. Ongoing and future Phase III trials will be essential to determine durability, safety and real‐world clinical benefit.

Comparative analyses place tirzepatide among the most effective pharmacological therapies for MASH, rivalling emerging agents such as FGF21 analogs and FXR agonists [11, 21]. Its inclusion in the 2024 EASL–EASD–EASO guidelines underscores its clinical relevance and growing acceptance in hepatic metabolic care [2, 28].

When citing guideline recommendations, we report both the strength of recommendation and the level of evidence as defined by each society. For example, the AASLD guidance provides a conditional recommendation (Strength: B) with moderate‐quality evidence (Level: II) for the use of GLP‐1–based therapies in patients with MASLD and coexisting obesity or T2D. Similarly, EASL guidelines classify incretin‐based therapies as having moderate evidence (Level B) supporting metabolic and hepatic benefits, while ADA guidelines issue a strong recommendation (Grade A) for their use in individuals with T2D and obesity due to robust glycaemic and weight‐loss efficacy. These classifications are now explicitly included throughout the manuscript to enhance clarity and transparency.

However, several gaps remain. Long‐term durability of response is uncertain, evidence in lean or nondiabetic MASLD is limited, and comparative data with liver‐targeted therapies are still lacking. Long‐term durability of histological response is unknown, as most data are limited to 52 weeks [15]. Evidence in nondiabetic, lean MASLD, or advanced fibrosis populations is limited [5, 10, 23]. Head‐to‐head comparisons with liver‐targeted agents are also lacking.

Overall, tirzepatide represents a major advancement in the management of MASLD/MASH, integrating metabolic control with meaningful hepatic improvements.

### 4.1. Research Gaps and Future Directions

Despite encouraging evidence, several important gaps remain. First, the long‐term durability of tirzepatide’s histological benefits is unknown, as most available data extend only to 52 weeks. Phase III trials with longer follow‐up are needed to determine whether improvements in steatohepatitis and fibrosis translate into reduced cirrhosis, hepatocellular carcinoma, and liver‐related mortality.

Second, evidence in specific subpopulations—such as lean MASLD, nondiabetic individuals, and patients with advanced fibrosis (F3–F4)—remains limited. These groups represent a growing proportion of MASLD cases and may respond differently to incretin‐based therapies.

To date, no major clinical trial has been specifically designed to evaluate tirzepatide in lean MASLD/NAFLD populations—individuals with hepatic steatosis despite a normal BMI and absence of obesity. This represents an important evidence gap, as lean MASLD may involve distinct metabolic and genetic drivers compared with obesity‐associated MASLD. Although tirzepatide may theoretically improve hepatic steatosis and inflammation through mechanisms partially independent of weight loss, dedicated trials are required to confirm these effects in lean individuals. Current trial registries do not list any ongoing studies targeting this subgroup, underscoring the need for future research to address this population.

Third, head‐to‐head comparisons between tirzepatide and liver‐targeted agents (e.g., FGF21 analogs and FXR agonists) are lacking. Combination therapy may ultimately prove superior, particularly for patients with advanced fibrosis.

Finally, real‐world data are needed to assess adherence, tolerability, and long‐term metabolic outcomes outside controlled trial settings. These insights will be essential for optimizing treatment strategies and identifying patients most likely to benefit.

## 5. Conclusion

Tirzepatide is a highly promising therapy for MASLD/MASH, offering robust metabolic improvements and significant hepatic benefits. Current evidence supports its integration into modern management strategies, although long‐term and phase III data are needed to confirm its disease‐modifying potential.

## 6. Limitations

This review also has limitations related to the available evidence base. Most clinical data for tirzepatide in MASLD/MASH derive from Phase II trials with relatively short follow‐up durations, which limits conclusions regarding long‐term histologic durability, progression to cirrhosis, or clinical outcomes such as decompensation and mortality. Additionally, no head‐to‐head trials comparing tirzepatide with other emerging metabolic or liver‐directed therapies (e.g., FGF21 analogs, FXR agonists, and GLP‐1 receptor agonists) are currently available. As a result, comparative interpretations rely on indirect evidence across heterogeneous study populations. Future Phase III trials with longer follow‐up and direct comparative designs will be essential to establish the relative efficacy and long‐term clinical impact of tirzepatide in MASLD/MASH.

## Author Contributions

Designed research, performed research: Angela Repanovici, Ileana Pantea. Analyzed data, data acquisition: Angela Repanovici. Writing – original draft: Ileana Pantea. Review and editing: All authors.

## Funding

No external funding was received for this work.

## Disclosure

All authors have read and agreed to the published version of the manuscript.

## Ethics Statement

The authors have nothing to report.

## Conflicts of Interest

The authors declare no conflicts of interest.

## Supporting Information

Additional supporting information can be found online in the Supporting Information section.

## Supporting information


**Supporting Information 1** Table S1. Top 10 most cited authors and contributing journals in tirzepatide–MASLD/MASH research (2018–2025). Table S1A. Top 10 most cited authors.

**Supporting Information 2** Table S1B. Top contributing journals.

## Data Availability

Data sharing is not applicable to this article as no datasets were generated or analyzed during the current study.
