# Supplementary material for: Tirzepatide in Metabolically Dysfunctional‐Associated Steatohepatitis (MASH): A Bibliometric and Evidence‐Based Review
Source: J Diabetes Res. 2026 Apr 10;2026:1853763. doi: 10.1155/jdr/1853763 (PMC13068985; doi:10.1155/jdr/1853763)
Supplement: Supplementary file 1 — Supporting Information 1 Table S1. Top 10 most cited authors and contributing journals in tirzepatide–MASLD/MASH research (2018–2025). Table S1A. Top 10 most cited authors. [file JDR-2026-1853763-s002.docx]

**Table S1. Top 10 Most Cited Authors and Contributing Journals in Tirzepatide–MASLD/MASH Research (2018–2025)**

**Table S1A. Top 10 Most Cited Authors**

| **Rank** | **Author** | **Affiliation** | **Main Contribution Area** |
| --- | --- | --- | --- |
| 1 | **Rohit Loomba** | UC San Diego, USA | MASH trials, fibrosis biomarkers, SYNERGY‑NASH |
| 2 | **Arun J. Sanyal** | Virginia Commonwealth University, USA | NASH pathogenesis, biomarkers, clinical trials |
| 3 | **Stephen Harrison** | Pinnacle Clinical Research, USA | Phase 2/3 MASH trials, antifibrotic therapies |
| 4 | **Michael Hartman** | Eli Lilly | Tirzepatide biomarker studies, incretin mechanisms |
| 5 | **David A. Lawitz** | Texas Liver Institute, USA | MASH clinical trials, fibrosis endpoints |
| 6 | **Giovanni Targher** | University of Verona, Italy | MASLD–cardiometabolic interactions |
| 7 | **Vlad Ratziu** | Sorbonne University, France | MASLD/MASH natural history and therapeutics |
| 8 | **Elisabet Bugianesi** | University of Turin, Italy | MASLD pathophysiology and clinical management |
| 9 | **Michael Trauner** | Medical University of Vienna | FXR agonists, bile acid metabolism |
| 10 | **Daniel Drucker** | University of Toronto, Canada | Incretin biology, GLP‑1/GIP mechanisms |
