# Supplementary material for: Tirzepatide in Metabolically Dysfunctional‐Associated Steatohepatitis (MASH): A Bibliometric and Evidence‐Based Review
Source: J Diabetes Res. 2026 Apr 10;2026:1853763. doi: 10.1155/jdr/1853763 (PMC13068985; doi:10.1155/jdr/1853763)
Supplement: Supplementary file 2 — Supporting Information 2 Table S1B. Top contributing journals. [file JDR-2026-1853763-s001.docx]

**Table S1B. Top Contributing Journals**

| **Rank** | **Journal** | **Publisher** | **Scope** |
| --- | --- | --- | --- |
| 1 | **Journal of Hepatology** | Elsevier | Liver disease, MASLD/MASH therapeutics |
| 2 | **Hepatology** | Wiley | Clinical trials, fibrosis, metabolic liver disease |
| 3 | **New England Journal of Medicine** | NEJM Group | Landmark clinical trials (e.g., SYNERGY‑NASH) |
| 4 | **Diabetes Care** | ADA | Incretins, T2D, metabolic biomarkers |
| 5 | **Diabetes, Obesity and Metabolism** | Wiley | Obesity pharmacotherapy, tirzepatide |
| 6 | **Gastroenterology** | Elsevier | MASLD mechanisms and therapeutics |
| 7 | **Metabolism** | Elsevier | Obesity pharmacology, metabolic pathways |
| 8 | **Expert Opinion on Investigational Drugs** | Taylor & Francis | Emerging MASH therapies |
| 9 | **World Journal of Gastroenterology** | Baishideng | MASLD pathophysiology and clinical updates |
| 10 | **J Cardiovascular Pharmacology and Therapeutics** | SAGE | Cardiometabolic implications of incretins |
